# Elasticity Mapping of Colloidal Glasses Reveals the Interplay between Mesoscopic Order and Granular Mechanics

**DOI:** 10.1002/smtd.202400855

**Published:** 2024-08-13

**Authors:** Thomas Vasileiadis, Marius Schöttle, Maximilian Theis, Markus Retsch, George Fytas, Bartlomiej Graczykowski

**Affiliations:** ^1^ Faculty of Physics Adam Mickiewicz University Uniwersytetu Poznanskiego 2 Poznan 61–614 Poland; ^2^ Department of Chemistry Physical Chemistry I University of Bayreuth Universitätsstr. 30 95447 Bayreuth Germany; ^3^ Bavarian Center for Battery Technology (BayBatt) Weiherstraße 26 95448 Bayreuth Germany; ^4^ Bavarian Polymer Institute (BPI) Bayreuth Center for Colloids and Interfaces (BZKG) Universitätsstraße 30 95447 Bayreuth Germany; ^5^ Max Planck Institute for Polymer Research Ackermannweg 10 55128 Mainz Germany; ^6^ Institute of Electronic Structure and Laser FORTH, N. Plastira 100 Heraklion 70013 Greece

**Keywords:** Brillouin light scattering, elasticity mapping, Gradient colloidal glasses, granular mechanics, self‐assembled polymer nanoparticles

## Abstract

Colloidal glasses (CGs) made of polymer (polymethylmethacrylate) nanoparticles are promising metamaterials for light and sound manipulation, but fabrication imperfections and fragility can limit their functionality and applications. Here, the vibrational mechanical modes of nanoparticles are probed to evaluate the nanomechanical and morphological properties of various CGs architectures. Utilizing the scanning micro‐Brillouin light scattering (µ‐BLS), the effective elastic constants and nanoparticles' sizes is determined as a function of position in a remote and non‐destructive manner. This method is applied to CG mesostructures with different spatial distributions of their particle size and degree of order. These include CGs with single‐sized systems, binary mixtures, bilayer structures, continuous gradient structures, and gradient mixtures. The microenvironments govern the local mechanical properties and highlight how the granular mesostructure can be used to develop durable functional polymer colloids. A size effect is revealed on the effective elastic constant, with the smallest particles and ordered assemblies forming robust structures, and classify the various types of mesoscale order in terms of their mechanical stiffness. The work establishes scanning µ‐BLS as a tool for mapping elasticity, particle size, and local structure in complex nanostructures.

## Graphical abstract

The two extremes of self‐assembled colloids are stiff perfect crystals and soft perfect glasses. But what happens in between? The answer is provided by elasticity mapping of a wide variety of colloidal glasses – continuous gradient structures, binary mixtures, and more – with scanning micro‐Brillouin light scattering. In this way, the various types of mesoscale order are classified according to their mechanical properties.

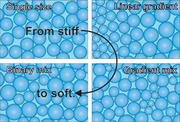

## 1 Introduction

The self‐assembly of colloidal nanoparticles can be harnessed for spatial modulations of the optical and mechanical properties toward controlling light and sound/heat propagation in photonic^[^ ^1^ ^]^ and phononic^[^ ^2^, ^3^ ^]^ crystals and metamaterials, respectively. Depending on the level of translational symmetry, we can describe the self‐assemblies between two extremes, that is, colloidal crystals (CCs) of ordered structure and colloidal glasses (CGs), characterized by the uncorrelated spatial disorder of the nanoparticles.^[^ ^4^, ^5^ ^]^

Engineering of light (photons) and sound/heat (phonons) propagation is possible both in amorphous and crystalline colloidal assemblies. In the former case, nanoparticles work as local resonators interacting with the waves propagating in the effective (air/vacuum‐solid) medium, leading to forbidden optical or acoustic (stop‐)bands. In CCs, the artificial periodicity results in multiple Bragg reflections of electromagnetic or acoustic waves, leading to bandgaps (Bragg) in addition to the disorder‐robust local resonance gaps.^[^ ^6^ ^]^ We note that this rigid categorization omits hyperuniform structures that reveal short‐range disorder (amorphous) accompanied by long‐range order (crystal).^[^ ^7^ ^]^ In parallel, colloidal glasses can be used to construct dye‐free structurally colored objects,^[^ ^8^, ^9^, ^10^ ^]^ and to optimize the thermal properties of thin glassy films.^[^ ^11^ ^]^

The interplay between glassy/amorphous and crystalline/periodic colloidal configurations is often used to acquire deep physical insights into the processes of glass transition^[^ ^11^, ^12^, ^13^ ^]^ and melting.^[^ ^11^ ^]^ Additionally, self‐assembled colloidal particles exhibit phenomena of granular mechanics,^[^ ^14^ ^]^ such as the jamming of soft particles.^[^ ^15^, ^16^, ^17^, ^18^, ^19^ ^]^ Colloidal assemblies are versatile model systems for studies of glass transition and jamming, as it is possible to explore different geometrical shapes and dimensionalities.^[^ ^20^ ^]^ In this respect, an exciting case of colloidal self‐assemblies is continuous gradient structures.^[^ ^21^ ^]^

A gradual spatial change of the particle size, colloidal mesostructure, or particle composition characterizes continuous gradient structures. We recently outlined how to fabricate such novel colloidal mesostructures and highlighted emerging new functionalities that arise from there.^[^ ^21^ ^]^ A gradual variation of the effective film‐forming temperature renders a colloidal crystal gradient into a useful time and temperature‐integrating sensor.^[^ ^22^ ^]^ Specifically, varying a binary particle size composition across the entire composition range sheds light on the transition between opalescence and strong light scattering in ordered versus disordered particle arrangements.^[^ ^23^ ^]^ This approach can be taken one step further when gradually varying particle size distributions are introduced. In such cases, ordered colloidal gradients or colloidal glasses with a broad but defined particle size distribution can be obtained. Both mesostructures demonstrated fascinating optical properties, particularly concerning efficient light scattering.^[^ ^21^ ^]^

As much as colloidal mesostructures strongly influence light propagation, the same should hold for acoustic waves of comparable wavelengths. This effect has been demonstrated for distinct colloidal mesostructures acting as phononic crystals.^[^ ^3^, ^24^ ^]^ In analogy to photonic crystals, phononic bandgaps have been demonstrated in well‐ordered polymer particle assemblies.^[^ ^25^ ^]^ Disturbing the crystalline order closes the Bragg gap but retains a stopband caused by the inherent particle‐matrix eigenmodes.^[^ ^26^ ^]^ Whereas the investigation of hypersonic bandgaps reveals the high‐frequency (GHz) elastic performance of colloidal assemblies of nanoparticles, the low‐frequency mechanical moduli are controlled by the colloidal arrangements. For instance, nanoindentation has been used to evaluate the influence of few‐layered colloidal crystals on their effective compressive strength.^[^ ^27^ ^]^

Generally, the transition from a crystalline to an amorphous (/glassy) configuration for a well‐defined particle diameter can expectedly weaken its effective elastic constant. However, we can now prepare a much larger variety of colloidal glasses with varying degrees of particle size dispersion and mesoscopic order using state‐of‐the‐art nanofabrication methods. The aim of this work is to understand how the elastic properties vary across the structural allotropes of colloidal glasses.

With the advent of micro‐Brillouin light scattering (µ‐BLS), a new tool has now become available to clarify the interplay between local mesostructure and mechanical properties in a much more rigorous way than contact‐based probes. This technique provides contactless, high‐resolution, direct, and non‐destructive insights into complex materials' local, high‐frequency mechanical properties. For instance, µ‐BLS can be used for momentum‐resolved measurements of hypersonic phononic crystals,^[^ ^6^, ^28^ ^]^ or in combination with temperature and pressure variations to study surface mobility^[^ ^29^ ^]^ and size‐dependent soldering^[^ ^30^, ^31^ ^]^ of polymer colloidal crystals.

Here, we extend the capabilities of µ‐BLS by combining it with a scanning sample stage for elasticity mapping of colloidal mesostructures with a micrometer spatial resolution. We employed µ‐BLS to investigate vibrational modes (GHz) of model colloidal assemblies such as crystals, glasses, gradient structures, and mixtures to highlight the effect of local order and nanoparticle size on the effective elasticity of the mesostructure.^[^ ^32^, ^33^ ^]^ We conclude our study with some basic rules governing the stiffness of colloidal assemblies, which hold across a wide range of prototypical colloidal systems.

## 2 Results and Discussion

### 2.1 Colloidal Glasses with Varying Mesoscopic Order

**Figure 1:**
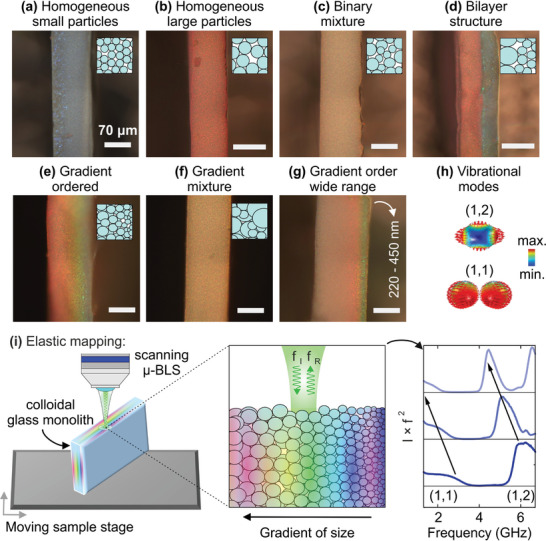
Optical microscopy images and micro‐Brillouin light scattering (µ‐BLS) experiment scheme. Cross‐section optical images of a) homogeneous small particles sample (HS, *D* = 220 nm), b) homogenous large particles sample (HL, *D* = 310 nm), c) binary mixture sample (BM, *D* = 220 nm and 310 nm), d) binary layered sample (BL, *D* = 220 nm and 310 nm), e) gradient ordered (GO, *D =* 220 – 310 nm), f) gradient mixed (GM, *D =* 220 – 310 nm), g) gradient ordered with a wide range of diameters (GOWR, *D* = 220 – 450 nm). i) FEM visualization of vibrational modes probed by BLS for mapping nanoparticles' sizes and effective elastic properties of colloidal assemblies: (1,2) quadrupolar and (1,1) translational spheroidal Lamb modes. The colors represent the magnitude of the displacement field, and the direction of displacement is shown with red arrows. h) Schematic illustration of scanning µ‐BLS experiments across the cross‐section of a colloidal glass monolith (left), BLS in backscattering geometry (center) where the incident frequency of light (f_I_) is changing after backscattering to (f_R_), and illustration of the BLS power spectra as a function of frequency (f = |f_I_‐f_R_|). The scale bars in (a–g) are 70 µm, and the insets show schematic illustrations of the mesostructures.

We examine seven types of colloidal glasses (CGs) whose optical microscopy images are shown in **Figure** **1**. The samples are self‐assembled from size‐selected poly(methyl methacrylate) (PMMA) particles with sub‐nm accuracy. We observe characteristic iridescent structural colors for all samples because the visible light wavelengths are comparable to the interparticle spacing. The samples are named according to their size and morphology characteristics as: 1) homogeneous small particles (HS); 2) homogeneous large particles (HL); 3) binary mixture (BM); 4) binary layered (BL); 5) gradient ordered (GO) with a linear increase of particle diameter as a function of position; 6) gradient mixed (GM); 7) gradient ordered with a wide range (GOWR) of diameters. The main difference between the GO and GOWR samples is that the latter has a non‐linear particle diameter increase as a function of position and features an extended particle size range.

Samples HS and HL (Figure 1a,b) consist of a single size of particles with diameters *D* = 220 nm and 310 nm, respectively. The sizes of nanoparticles were obtained utilizing dynamic light scattering (DLS). Sample BM is an amorphous binary mixture of nanoparticles of these two sizes (Figure 1c) with a 50/50 wt% composition. Sample BL is a bilayer structure prepared by depositing a layer of the large particles (310 nm) first and then adding a layer of small (220 nm) particles on top (Figure 1d). The sample GO is a gradient colloidal assembly in which the diameter linearly increases from right to left in Figure 1e within the 220–310 nm range. The sample GM (Figure 1f) has the same range (220‐310 nm) of *D* but it is randomly mixed. Finally, sample GOWR is also a gradient colloidal assembly where the particle diameter continuously increases from right to left in Figure 1g in the *D* range 220–450 nm. Details for the fabrication of all these colloidal glass mesostructures are given in the work of Schöttle et al.^[^ ^21^ ^]^

### 2.2 Elastic Mapping with Scanning µ‐BLS

The local elastic properties and particle size of each sample have been probed through the (1,1) and (1,2) spheroidal Lamb modes (Figure 1h) with µ‐BLS in backscattering geometry (see Figure 1i and Experimental Section). With the aid of a scanning sample stage, µ‐BLS could record the spatially resolved acoustic spectra of the nanoparticles across the cross‐section of the colloidal glasses with 1 µm steps (Figure 1i) and spatial resolution of ≈ 2.6 µm (S1, Supporting Information). The colloidal glass monolith cross‐sections (shown in Figure 1a–g) were parallel to the scanning µ‐BLS stage. The scanning direction (black arrow in Figure 1i) was parallel to the direction of particle size gradients. For each measurement, the raw spectra were normalized by the intensity of the elastic peak (at zero frequency) and multiplied by *f* ^2^, the thermal occupation factor for phonons of frequency *f*.^[^ ^29^, ^34^ ^]^ In this way, the power spectra are proportional to the vibrational density of states modulated by the BLS activity of each mode.

**Figure 2:**
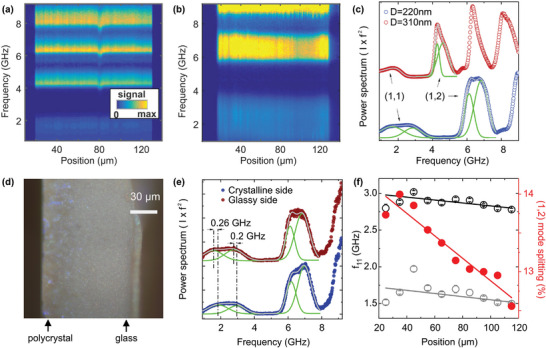
Scanning µ‐BLS of homogeneous size colloidal assemblies. Spatial distribution of BLS signal (power spectrum) a) for the sample HL with large particles (*D* = 310 nm) and b) for the sample HS with small particles (*D* = 220 nm), respectively. c) The power spectra for the large and small particles averaged over the entire structure (circles) and the representation (green solid lines) with asymmetric‐double sigmoidal functions (large particles) and double Gaussian functions (small particles) for the (1,1) mode, and double Gaussian functions for the (1,2) mode. d) The optical microscopy image with arrows indicating the crystal‐to‐glass transition in HS. e) The power spectra on the crystalline (blue points) and glassy (red points) sides of HS and their representation (solid grey line) with Gaussian functions (green lines). The vertical dash‐dot lines indicate the redshift of the *f*_11_ mode in the glass compared to the crystal. f) The *f*_11_ for the two spectral components of the (1,1) mode, and the relative splitting of (1,2) mode in HS as a function of the spatial position (points) and their representation with linear functions (solid lines).

**Figure** **2**a,b shows spatially resolved µ‐BLS power spectra of single‐size colloidal assemblies, HL and HS with *D* = 310 nm and 220 nm, respectively. Additionally, Figure 2c displays the µ‐BLS spectra of both samples averaged over the measured cross‐section. For the HL sample, the first low‐frequency peak, centered at *f*_11_ ≃ 1.8 GHz, was assigned to the translational (1,1) Lamb mode; notably, the (1,1) mode is of zero frequency for individual or non‐interacting nanoparticles. In the case of interactions via mechanical contacts, the (1,1) mode can be resolved by BLS as an asymmetric peak resembling the phonic density of states (DOS).^[^ ^34^ ^]^ The spectral position of this peak reflects the magnitude of the interparticle bondings and can be translated into the effective elastic modulus of the colloidal assembly. The (1,1) mode for the large particles can be represented with an asymmetric‐double‐sigmoidal (asym2sig) function with a peak position at 1.64 GHz. The (1,2) mode is a double Gaussian, and its splitting into two frequencies ($f_{12}^{\prime }$= 4.27 GHz and 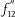
= 4.62 GHz) due to interactions (contacts) with the nearest neighbors, thereby lifting the degeneracy of the mode^[^ ^29^, ^30^, ^34^ ^]^ The peaks above 6 GHz correspond to higher‐order BLS‐active spheroidal modes.

For the HS sample made of 220 nm particles, the BLS peaks of the (1,2) mode are blueshifted compared to the large particles by the diameter ratio (≈ 1.4). The (1,1) mode (Figure 2c) of the small particles (*D* = 220 nm) is better represented by a double Gaussian at 1.64 and 2.84 GHz. The (1,2) mode is again represented by a double Gaussian at $f_{12}^{\prime }$ = 6.13 and $f_{12}^{\prime \prime }$ = 6.83 GHz. From these frequencies, we can calculate the reduced frequency, *f_r_* = 2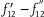, which for the known particle diameter yields the transverse sound velocity,^[^ ^25^ ^]^ *c_T_*:

(1) $$c_{T}=\frac{Df_{r}}{A}$$

where *A* is a constant equal to 0.84 for spheres made of materials with Poisson's ratio in the range of 0.16‐0.32,^[^ ^35^ ^]^ and *c_T_* =  1420 m s^−1^ for bulk PMMA.^[^ ^36^ ^]^ From Equation (1), we extract *D* = 219 and 303 nm for the small and big particles, respectively, in agreement with the DLS measurements.

**Figure 3:**
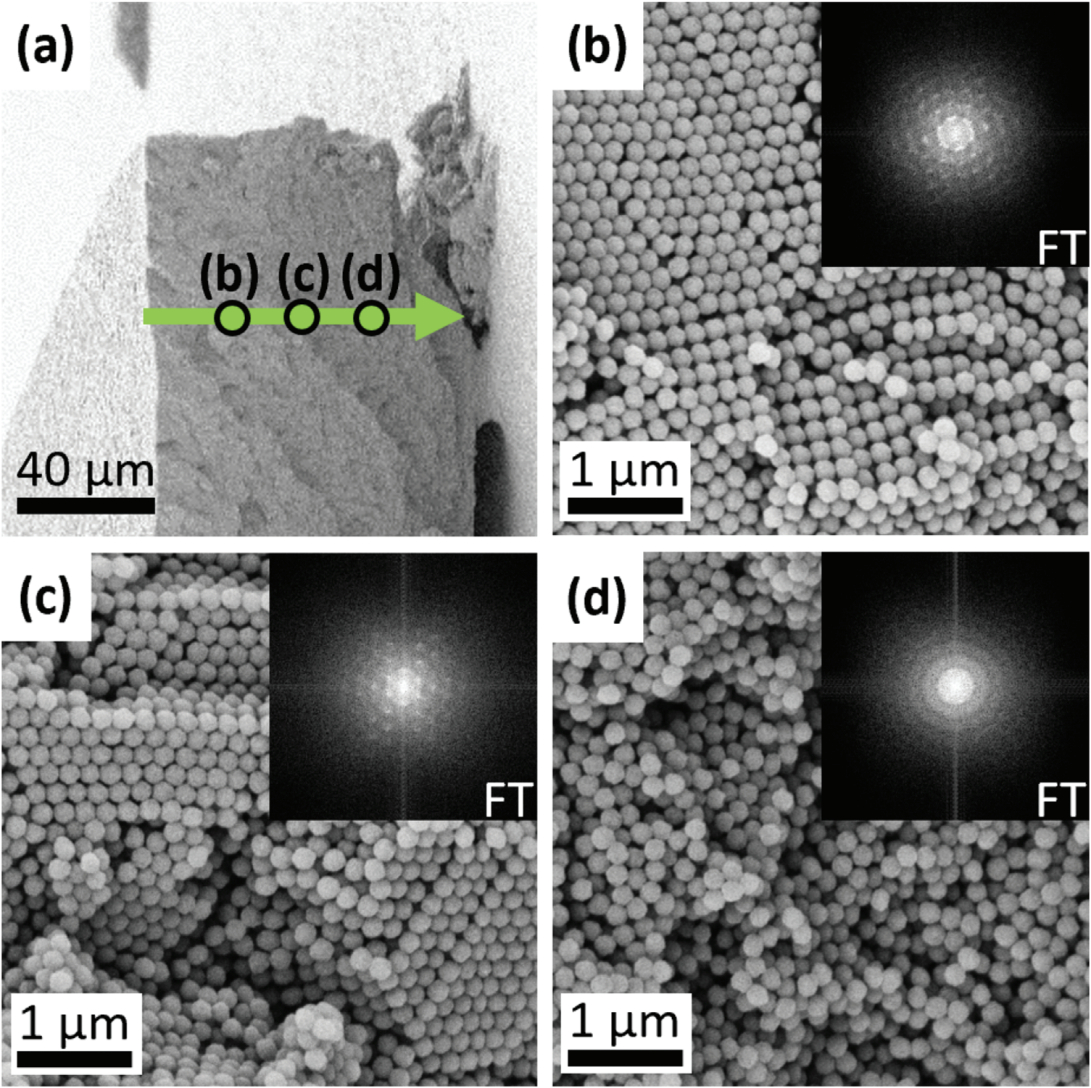
SEM imaging of crystal‐to‐glass transition. a) SEM image at low magnification of the cross‐section of sample HS. The green line in (a) indicates the direction along which SEM images at higher magnification capture the particle order close to the polycrystalline surface b), approximately at the middle c), and close to the glassy surface d).

Next, we examine the spatially resolved spectral line shapes of the HS sample to identify structural inhomogeneities and variations of the elastic properties. This analysis revealed that – contrary to the behavior of larger particles – the smallest particles with D = 220 nm in the HS sample assume partially crystalline configurations. We first notice that the optical microscopy image of the HS sample displays a crystal‐to‐glass transition through its cross‐section. The black arrows in Figure 2d indicate a polycrystalline region on the left edge characterized by a rough surface due to grain boundaries and a homogeneous glassy region on the right edge, with a spatially smooth transition between the two phases. This structure is confirmed by an imaging scan with SEM along the cross‐section of sample HS, which is shown in **Figure** **3**. At approximately the center of the HS sample, the SEM images and their Fourier Transform (FT) show a partially polycrystalline structure. The observed structure becomes purely amorphous close to the right edge of the HS sample, and the FT shows only rings. For additional characterization of the HS sample with SEM, see Figure S2 (Supporting Information). Apart from the HS sample and the small‐particle side of the BL sample (also D = 220 nm), all other colloidal assemblies containing larger particles showed no sign of long‐range order. For the SEM characterization of the particle assemblies, see ref. [21] and its (Figures S10,S16, and S17, Supporting Information).

The inhomogeneities observed with optical microscopy on the one side of the HS sample (Figure 2d) are attributed to the existence of crystalline domains. The other side of the sample appears to be homogeneous and amorphous (glassy). However, in contrast to the optical impression provided in Figure 2d, the spectroscopic analysis of Figure 2f does not show any sudden change in the colloidal order but a gradual transition from disordered amorphous regions to a polycrystalline arrangement. This gradual loss of crystallinity can be explained by the fabrication process. For all samples shown here we used vacuum filtration. The observed glass‐to‐crystal transition stems from the hampering of convective flow as the colloidal glass grows thicker. In more detail, the particles preferentially crystallize when experiencing low flux. The particles deposited first on the filter membrane experience the highest flux and, consequently, arrange in a glassy state when the colloidal glass monolith starts forming. As the filtration progresses and the colloidal monolith grows in thickness, the flux decreases and enables the formation of more crystalline structures.

Figure 2e shows the experimental power spectra (points) on the crystalline (blue) and glassy (red) edges of the HS sample, along with their representation (solid lines) with Gaussian peaks. Noticeably, the frequencies of the two (1,1) components are redshifted by ≈ 0.2‐0.26 GHz in the glass compared to the crystal, as indicated by vertical dash‐dot lines. For the crystalline state, these frequencies amount to 1.83 and 2.95 GHz, and for the glassy state, 1.56 and 2.80 GHz. Figure 2f depicts the drop of these two frequencies with the distance from the polycrystalline to the glassy edge for HS with small particles (*D* = 220 nm). The drop is resembled by a linear fit with −0.067%/µm slope for the high‐frequency component and −0.14%/µm for the low‐frequency component of (1,1) mode. The thickness of the HS film is 110 µm, suggesting a ≈7% and 15% difference in the high and low f_11_ frequencies of the crystalline and glassy edges, respectively. The observation that the f_11_ frequencies drift across the HS cross‐section has been verified with additional measurements (Figures S3 and S4, Supporting Information). For the (1,2) mode in HS, the frequencies are 6.19 and 6.98 GHz in the crystalline and 6.12 and 6.84 GHz in the glassy phase, with an experimental error of ≈ 1.5%.

The relative split of the (1,2) mode, expressed as 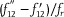 is also shown (red symbols) in Figure 2f. It drops from the crystalline to the glass edge with −0.011%/µm slope. Since the (1,2) mode split reflects the contact strength between particles (it becomes zero in the absence of contacts), it provides an additional measure of a crystal‐to‐glass transition and softening of interparticle bonds. Noticeably, the observed frequency shifts are linearly changing across the entire sample, corroborating the already discussed continuous change from polycrystallinity to a glassy state. For the small particles (sample HS), both the blueshift of the (1,1) mode components and the split of the (1,2) mode suggest that the PMMA particles form stronger contacts on the crystalline side of the sample. Weaker or fewer contacts between nanoparticles can explain the gradual softening of the glass. In contrast, the HL sample, which consists only of large particles with purely glassy configurations, does not show any discernible spatial inhomogeneities of the (1,1) mode (Figure S5, Supporting Information).

The position‐dependence of *f*_11_ in HS (Figure 2f) is attributed to the spatial crystal‐to‐glass transition shown in Figure 2d, with the crystalline phase possessing the highest frequency. The *f*_11_ peak has a high‐frequency component that is attributed to longitudinal acoustic phonons, and it can be used to extract the effective elastic modulus^[^ ^30^, ^31^, ^34^ ^]^ (*E*_eff_) from:

(2) $$E_{\mathrm{eff}}=\frac{1}{2}\;p_{\mathrm{eff}}\pi^{2}D^{2}f_{11}^{2}$$

where *p*_eff_ =  *a* *p* + (1 − *a*) · 1.225, is the effective density of the colloidal assembly, and *p*  =  1180 kg m^−3^ is the polymer density and 1.225 kg m^−3^ of the air at ambient conditions. The particle volume fraction amounts to *a* = 0.74 for crystalline assemblies, while for glassy configurations with close random packing^[^ ^37^, ^38^ ^]^ *a* ≈  0.65, which also applies to samples with size polydispersity.^[^ ^39^ ^]^ Due to the changes in density and the dependence of *f*_11_ frequency on nanoparticle contacts, the quantity *E*_eff_ is sensitive to crystal‐to‐glass transitions. With Equation (2) and the experimental data of Figure 2e, we obtain *E*_eff_ =  1.82 GPa for the crystal and *E*_eff_ =  1.43 GPa for the glass, that is, ≈ 20% stiffer crystalline phase. Note that the Young modulus of bulk PMMA amounts to ≈ 6.2 GPa.^[^ ^40^ ^]^

Another pertinent observation regarding the spectral shape of the (1,1) mode is its double peak structure. The signal of the low‐frequency component, which is mainly located in the 1–1.5 GHz region, is enhanced close to the edges of the HS sample (Figure S6, Supporting Information). The low‐frequency component of the (1,1) mode can originate from transverse acoustic waves in the CG structure that become BLS active after multiple light scattering in backscattering. In agreement with this interpretation, the spectral shape of *f*_11_ resembles the calculated vibrational density of states of an FCC lattice made of *D* = 220 nm PMMA spheres with an effective spring constant^[^ ^34^ ^]^ of $K_{\mathrm{eff}}=\pi^{2}\;M\;f_{11}^{2}=\;565$ N/m (Figure S7, Supporting Information), where *M*  = 4π *p* (*D*/2)^3^/3 is the particle mass as a function of the particle diameter.

### 2.3 Elasticity in Spatially Inhomogeneous Glasses

**Figure 4:**
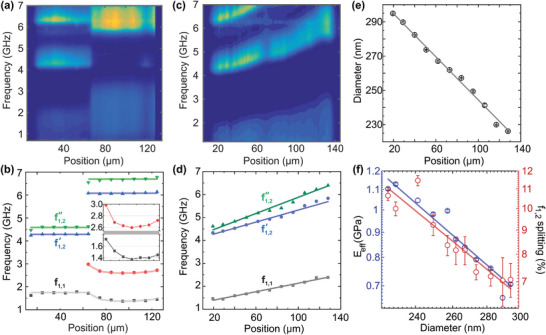
Scanning µ‐BLS of bilayer and gradient CGs. a) Spatial distribution of the power spectrum for the bilayer sample BL. b) The frequencies *f*_11_ (red and grey), $f_{12}^{\prime }$ (blue), and 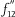 (green) for the bilayer structure. The interface is located at 75.5 µm. Inset: the spatial evolution of the frequencies *f*_11_ (red and grey) for the small size layer. c) Spatial distribution of the power spectrum for the gradient GO structure. d) The frequencies *f*_11_ mode (grey), $f_{12}^{\prime }$ (blue), and 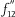 (green) for the gradient structure. e) The particle diameter as a function of the position for the gradient GO structure. **(f)** Log‐log plot of *E*_eff_ as a function of *D* for the GO gradient (blue points) and a fitted linear function (solid line) with slope −1.9 ± 0.2.

The spatial distribution of the BLS spectra for the bilayer BL sample is shown in **Figure** **4**a. The BLS spectra are consistently represented by asym2sig functions for the (1,1) of the large particles, double‐Gaussians for the (1,1) of the small particles, and double‐Gaussians for the (1,2) mode of both layers. The extracted position‐dependent frequencies are shown in Figure 4b. From $f_{12}^{\prime }$ and 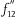 and Equation (1), the particle diameter in the large (/small) particle side is 300 ± 4 nm (/218 ± 1 nm), which is expectedly in agreement with the size in HL (/HS). Notably, the (1,1) mode of the small particles' side shows again a spatial modulation of its two components (inset to Figure 4b). The (1,1) frequencies are higher at the small particle layer and at the interface. The observed higher frequencies of (1,1) are in agreement with the results of Figure 2 and the optical images of the bilayer sample (Figure 1d) that show crystalline regions in the small particles' edge. Moreover, the interface of the bilayer structure, which is characterized by uncertainty in the particle size, appears to have a higher *f*_11_ frequency, suggesting a stiffer local binding between particles. In accordance with this result, the SEM images of the BL interface in Figure S8 (Supporting Information) clearly show the formation of extended crystalline domains on the small particle side.

In contrast to the abrupt particle size change in the BL sample, an incremental particle size variation is realized in the sample GO. The power spectra map for the GO sample is shown in Figure 4c, and the deduced $f_{12}^{\prime }$ and 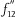 frequencies (Figure 4d) are used to extract the position‐dependent particle diameter (black circles in Figure 4e) with an average error in the order of 1% (S5, Supporting Information). The position‐dependent diameter can be represented with a linear decrease of slope −0.64 ± 0.02 nm/µm (solid line in Figure 4e). From the experimental data for f_11_ of Figure 4d and Equation (2), we find the size (position)‐dependent effective elastic constant for the CG gradient GO sample (Figure 4f). Noticeably, the smaller particles display stronger binding and form stiffer CGs (see Figure S11, Supporting Information for all samples). The representation of the experimental data on the log‐log plot of Figure 4f (points) with a linear function (solid line) yields a scaling behavior, *E*_eff_∝*D* ^−1.9^, which according to Equation (2) approximately implies *f*_11_∝*D* ^−2^. Note that *E*_eff_(*D*) is much stronger than the *E*_eff_∝*D* ^−1/3^ obtained from the scaling *f*_11_∝*D* ^−7/6^ in the framework of the JKR model.^[^ ^41^ ^]^ The stronger binding of the smaller particles is also reflected in the increased splitting of $f_{12}^{\prime }$ and 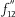 with decreasing particle size in Figure 4d. The larger splitting of $f_{12}^{\prime }$ and 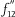 for smaller particles is also clearly visible in the results of Figure 4b.

### 2.4 Spatially Homogeneous Bidisperse and Polydisperse Glasses

**Figure 5:**
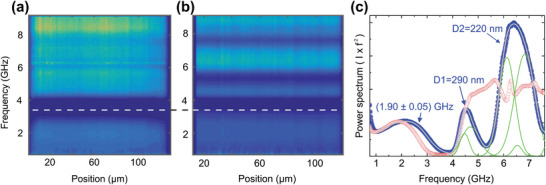
Scanning µ‐BLS of mixed gradient and mixed binary CGs. Spatial distribution of the power spectrum for the mixed gradient GM sample a) and the mixed binary BM b) samples. The dashed lines in a) and b) serve as a comparison of the frequency range of the (1,1) mode in the GM film relatively to the BM. c) Power spectra averaged over the entire cross‐section for the mixed binary BM (blue circles) and the mixed gradient GM sample (red circles). The spectrum of BM is represented with an asym2sig function for the (1,1) mode and Gaussian peaks (green solid lines) for the sphere modes.

The observation that smaller particles show higher effective elastic constant – derived from the gradient ordered glass – can now be compared to the properties of the spatially homogeneous, glassy systems with size polydispersity: the gradient mixed GM and the binary mixed BM samples whose spatially resolved power spectra are shown in **Figure** **5**a,b, respectively. From the visual inspection of the maps, the binary mixture appears to have a blueshifted (1,1) mode compared to the gradient mixed, as shown by a horizontal dashed line, for comparison.

Figure 5c shows the power spectra averaged over the entire cross‐section for the mixed binary BM (blue circles) and the mixed gradient GM sample (red circles). In the spectrum of BM, the (1,1) mode is located at 1.9 GHz, while the (1,2) mode appears at 4–5 GHz for the large particles (*D* ≈ 300 nm), and at ≈6‐7 GHz for the small particles (*D*  = 220 nm). For an accurate representation of the spectral lineshape, a weak Gaussian peak is added at ≈6.5 GHz, which can be attributed to the higher‐order sphere mode of the large particles. Noticeably, the area of the (1,2) mode is larger for the small particles (Figure 4c) by a factor of almost 5 compared to that of large particles. For 50% mixing per mass means there are more than twice (2.3) small particles per volume. For comparison, Figure 5c also shows the average power spectrum of the gradient mixed GM sample (red circles). In this sample, the (1,1) mode is redshifted, compared to BM, to 1.78 GHz (Figure S12, Supporting Information). The signal in the ≈4–6 GHz region is due to the (1,2) modes of a continuum of nanoparticle sizes from *D*  = 310 nm (*f_r_*  = 3.93 GHz) to *D*  = 220 nm (*f_r_* = 5.54 GHz); the narrow peak at ≈6.5 GHz is a laser sideband, and higher‐order spheroidal Lamb modes can explain the signal above 6.5 GHz.

From the GM sample preparation, the average diameter (volume fraction) is estimated to be <*D*> = 257 nm and the (1,2) mode would have occurred at ≈ 5.3 GHz, close to the quasi‐peak in Figure 5c. The effective elastic moduli are approximately equal, 0.81 ± 0.02 GPa for GM and 0.79 ± 0.02 GPa for BM with an average diameter of 243 nm. The difference is in the order of ≈2%, while the scaling *E*_eff_∝*D* ^−1.9^ (derived from the gradient‐ordered glass) would predict ≈10%. In contrast, the gradient‐ordered glasses show a strong size dependence (Figure S11, Supporting Information). As the filling factor (*a*), which is used to calculate *E*_eff_, depends on the level of polydispersity, we have also extracted the effective spring constant *K*_eff_. For BM with <*D*> = 243 nm, *K*_eff_ = 316 ± 16 N/m versus 328 ± 18 N/m for GM with <*D*> = 257 nm, oppositely to what is expected by the size trend, that is, smaller particles imply stronger binding. The effective elastic moduli and spring constants for all samples are summarized in **Table** **1**. In the case of gradient‐ordered samples, we compare the values of *E*_eff_ and *K*_eff_ for selected average diameters (meaning for specific points on the sample). The comparisons of *E*_eff_, have been repeated for a range of filling factors: *a*  =  0.6‐0.74, and the results are shown as error bars in S7 (Supporting Information).

**Table 1:** Summary of effective elastic moduli and spring constants for all the samples.

Sample	Range and local value of *D* (nm)		*f*_11_ [GHz]	*E*_eff_ [GPa]	*K*_eff_ [N/m]
HS glass	220		2.80	1.44	509
HS crystal			2.95	1.82	565
HL	310		1.64	0.94	462
BL small	220		2.7	1.33	471
BL large	310		1.72	1	486
GOWR	220‐450 (gradient)	≈310	1.35	0.65	322
GO	220‐310 (gradient)	≈243	2.17	1.04	402
GM	220‐310	257	1.78	0.79	328
BM	220 & 310	243	1.9	0.81	316

## 3 Conclusion

We have prepared a large variety of prototypical colloidal glasses, all from the same material and with the same basic synthesis, where only the particle mesoscopic structure varied. This is quite a different approach than the engineering of materials' mechanical modulus, where gradients have mainly been introduced by changing the cross‐linking density. We have demonstrated efficient elastic mapping of various types of CGs, including novel gradient structures, using scanning µ‐BLS. The µ‐BLS measurements show how the structure, size, and elastic properties evolve in the space of complex colloidal glassy systems, which cannot be derived from optical or electron microscopy images. Contrary to electron microscopy, which can damage the molecular structure and change the nanoparticles' morphology, µ‐BLS employs visible radiation well below the energy gap of the polymer. Moreover, µ‐BLS is a contactless, non‐destructive technique that cannot trigger jamming transitions like force‐based techniques.^[^ ^42^ ^]^

Noticeably, µ‐BLS of gradient‐ordered glasses indicated a size effect, where smaller particles form stiffer CGs. Although a particle size effect is qualitatively expected from the JKR model,^[^ ^41^ ^]^ the observed size dependence is much stiffer than the JKR prediction, with *E*_eff_ and *f*_11_ being roughly proportional to ≈ *D* ^−2^. This effect can arise from various microscopic features of CGs.

From the perspective of the Hertzian contact theory, the particles initially attract each other by van der Waals (vdW) forces but deform upon contact, creating a Hertzian contact area (a flattened interface) between touching spheres. Based on thermal conductivity measurements of polystyrene‐silica core‐shell colloidal crystals, the contact area (normalized to the particle surface area) is known to increase with a decrease in the particle size^[^ ^43^ ^]^ in the submicrometer range. Hence, the larger contact area of the smaller particles can rationalize a higher *E*_eff_.

Additionally, the interstitial space can be filled by free polymer chains or surfactants, which are inevitable side‐products of the emulsion polymerization process. Due to capillary forces, this excess material will be immobilized at the interparticle contact points and enhance *E*_eff_.^[^ ^44^, ^45^ ^]^ Analogously to the evolution of Hertzian contact areas, the smaller the particle size, the larger the enhancement of *E*_eff_ for a given density of free polymer chains.^[^ ^31^ ^]^

Moreover, the structures of glasses contain voids and regions with a different short‐range order than the corresponding equilibrium crystal, e.g., entirely FCC, BCC, or simple cubic. The voids and short‐range order variations can affect the density and the nearest neighbor number, the *f*_11_, *f*_12_‐splitting, and finally *E_eff_*. Intermediate‐range order effects are known to play an important role in colloidal supercooled liquids, especially for bidisperse colloids. In this context, the BM and GM samples appeared to be softer than regions of the GO sample of the same particle size, while the homogeneous monodisperse samples appeared stiffer – see Table 1.

Monodisperse^[^ ^46^ ^]^ or polydisperse^[^ ^47^ ^]^ colloids form glass‐like solids through jamming. Our results indicate that jammed glasses become softer by introducing a linear gradient order and soften even further with spatially homogeneous size polydispersity. Thus, softening involves both long‐range phenomena (i.e., the existence of a gradient order), and nearest‐neighbor interactions (i.e., the short‐range order). For applications requiring durability, polydisperse or gradient‐ordered glasses may need to be post‐treated to enhance their stiffness using for instance: UV radiation, thermal treatment,^[^ ^29^ ^]^ or pressurization with supercritical fluids.^[^ ^30^, ^31^ ^]^ The methods reported here pave the way for designing, characterizing, and optimizing complex colloidal glasses for robust or morphable devices.^[^ ^8^, ^48^ ^]^

## 4 Experimental Section

The colloidal mesostructures were prepared as outlined in detail in a recent work.^[^ ^21^ ^]^ In short, emulsion polymerization was used to synthesize PMMA seed particles. The controlled emulsion extraction process (CrEEP) was used to access gradient particle size distributions. The binary mixture was prepared from monodisperse dispersions. The colloidal mesostructures were fabricated by a vacuum filtration process using the required particle dispersion. After drying, freestanding colloidal superstructures with a thickness in the range of 100–150 µm were obtained.

Scanning Electron Microscopy (SEM) images were obtained with a Zeiss LEO 1530 (Carl Zeiss AG, Germany) at an operating voltage of 3 kV, 30 µm aperture, and an Everhart‐Thornley SE2 detector.

The wavelength of light used for µ‐BLS was λ = 532 nm, emerging from a single‐mode CW laser (Spectra‐Physics, Excelsior 300), and we employed the backscattering geometry. In backscattering geometry from bulk samples, the momentum transfer is q = 4πn/λ, where n is the refractive index, while the incident frequency of light (f_I_) is changing to f_R_ with f = |f_I_‐f_R_| being the phonon frequency. For samples consisting of nanospheres, the information about momentum is lost due to multiple scattering. For this geometry, we used a R:T,45:55 pellicle beam splitter. For the samples examined here, the vibrational modes are non‐dispersive (confined sphere modes), as the scattered light momentum is ill‐defined due to multiple scattering. The incident‐scattered light polarization was s‐s (also termed VV, TE‐TE). The inelastically scattered light was analyzed with a Fabry–Perot interferometer (JRS Scientific Instruments). The mirror spacing was 15 mm and the scan amplitude was 490 nm (FSR ±9.2 GHz), while the entrance (/exit) pinhole was 350 µm (/450 µm).

## Conflict of Interest

The authors declare no conflict of interest.

## Supporting information

Supporting Information

## Data Availability

The data that support the findings of this study are available from the corresponding author upon reasonable request.
