# Supplementary material for: Elasticity Mapping of Colloidal Glasses Reveals the Interplay between Mesoscopic Order and Granular Mechanics
Source: Small Methods. 2024 Aug 13;9(3):2400855. doi: 10.1002/smtd.202400855 (PMC11926515; doi:10.1002/smtd.202400855)
Supplement: Supplementary file 1 — Supporting Information [file SMTD-9-2400855-s001.pdf]

small methods

Supporting Information

for *Small Methods*, DOI 10.1002/smtd.202400855

Elasticity Mapping of Colloidal Glasses Reveals the Interplay between Mesoscopic Order and Granular Mechanics

Thomas Vasileiadis, Marius Schöttle, Maximilian Theis, Markus Retsch, George Fytas* and Bartlomiej Graczykowski**

Supporting Information: Elasticity mapping of colloidal glasses reveals the interplay between mesoscopic order and granular mechanics

Thomas Vasileiadis[1]*, Marius Schöttle[2], Maximilian Theis[2], Markus Retsch[2,3], George Fytas[4,5]*, Bartlomiej Graczykowski[1,4]*

[1] Faculty of Physics, Adam Mickiewicz University, Uniwersytetu Poznanskiego 2, 61-614 Poznan, Poland
[2] Department of Chemistry, Physical Chemistry I, University of Bayreuth, Universitätsstr. 30, 95447, Bayreuth, Germany
[3] Bavarian Center for Battery Technology (BayBatt), Weiherstraße 26, 95448 Bayreuth, Germany; Bavarian Polymer Institute (BPI); Bayreuth Center for Colloids and Interfaces (BZKG), Universitätsstraße 30, 95447 Bayreuth, Germany
[4] Max Planck Institute for Polymer Research, Ackermannweg 10, 55128 Mainz, Germany
[5] Institute of Electronic Structure and Laser, FORTH, N. Plastira 100, Heraklion, 70013 Greece

* thomas.vasileiadis@amu.edu.pl

* fytas@mpip-mainz.mpg.de

* bartlomiej.graczykowski@amu.edu.pl

S1. Spatial resolution of μ-BLS

To measure the spatial resolution of μ-BLS we use the spatial dependence of the intensity of the (1,1) mode across the interface of the bilayer sample BL (**Figure S1** up), and represent it with the convolution of a Gaussian with a step function, which is given by:

$$C(f) = \frac{A}{2}\left[1 - \mathrm{erf}\left(\frac{x - x_o}{\sqrt{2}\sigma}\right)\right] \quad ,$$

where x_o is the position of the interface and σ the width of the Gaussian. The σ=1.1 μm (FWHM=2.6 μm), which is expectedly close to the estimated focus spot diameter given by 1.22·λ/N_A=1.6 μm (λ the wavelength and N_A=0.4 the numerical aperture of the x50 microscope objective). The same trend, albeit more noisy, is observed for the signal of the (1,2) modes of the large particles (**Figure S1** down).

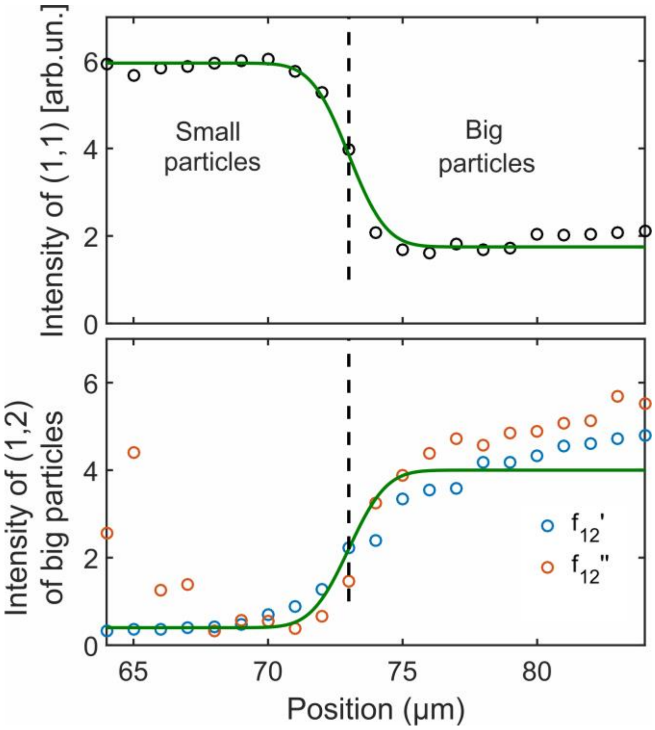

Figure S1 Up: Intensity of the (1,1) mode at the interface of the BL sample (points) vs. representation with the convolution of a Gaussian and a step function (green line). Down: The same convolution function plotted together with the (1,2) mode intensities of big particles (points).

S2. Additional information on single-sized HS (small particles) and HL (big particles)

Figure S2 shows a detailed characterization with SEM of the crystal-to-glass transition in the HS sample, which is homogeneous in terms of size and consists of the smallest particles (220 nm diameter).

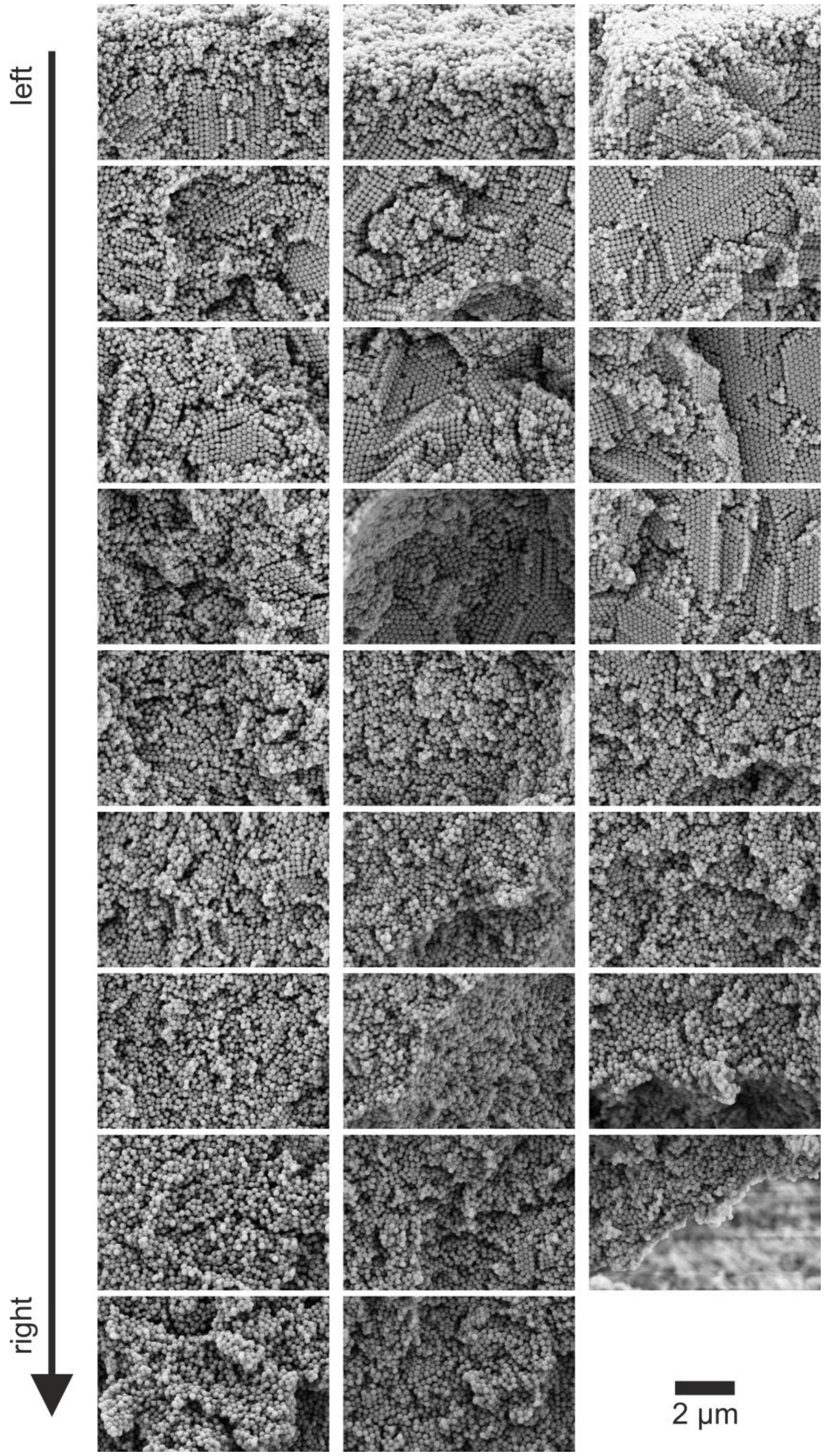

Figure S2: Detailed SEM images of the gradual transition from the polycrystalline regions of HS to the glassy parts.

Figure S3a shows the experimental power spectra and **Figure S3b** the spectral shapes (points) of the (1,1) mode for the small PMMA particles at distances 23 µm (red) and at 122 µm (blue) and their representations (solid lines) with two Gaussians at high (dashed) and low (dot) frequency. Noticeably,

moving from 23 to 122 µm results in a blueshift of the entire spectrum and both Gaussian components. **Figure S3c** displays the frequencies $f_{1,1}$ of the two spectral components versus position (points) along with linear representations with slope -(12 ± 4)·10^{-4} GHz/µm for the high-frequency component and slope -(18 ± 7)·10^{-4} GHz/µm for the low frequency component. Expressed in relative change, the slope is 0.041%/µm and 0.11%/µm, respectively the high and low frequency components. The measurement has been repeated on a different sample spot with higher spectral resolution and the resulting slope of the f_{11} is shown in **Figure S4**.

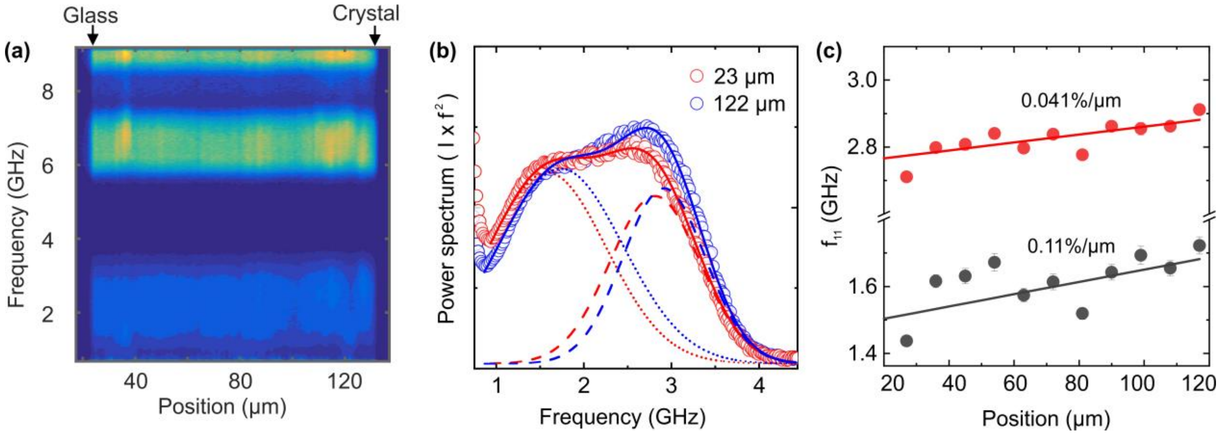

Figure S3: Additional measurement of HS. **(a)** Spatial distribution of BLS signal (power spectrum) for the sample HS with small particles (D=220 nm). **(b)** Comparison of the power spectra of the (1,1) mode for the HS sample (D=220 nm): at 23 µm (red) and at 122 µm (blue) with 8 µm binning. **(c)** The spectral positions of (1,1) components as a function of the spatial position (points) and their representation with linear functions (solid lines).

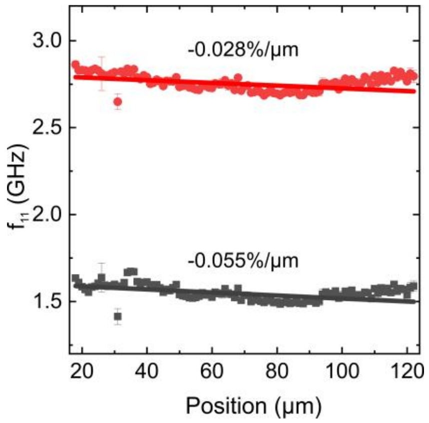

Figure S4: Measurement on a different spot of HS of the spectral positions of (1,1) components as a function of the spatial position (points) and their representation with linear functions (solid lines) for measurement with free spectral range ±5 GHz.

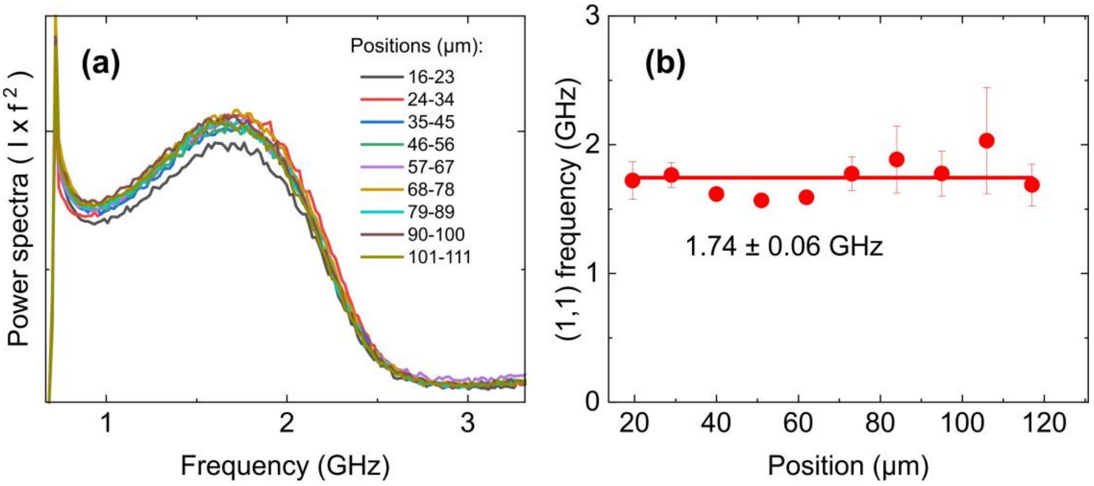

Figure S5: (a) The (1,1) mode for the single-size sample HL sample with large particles (D=310 nm). **(b)** The fitted f11 versus position (points) yields an average frequency of 1.74 GHz marked by the solid line.

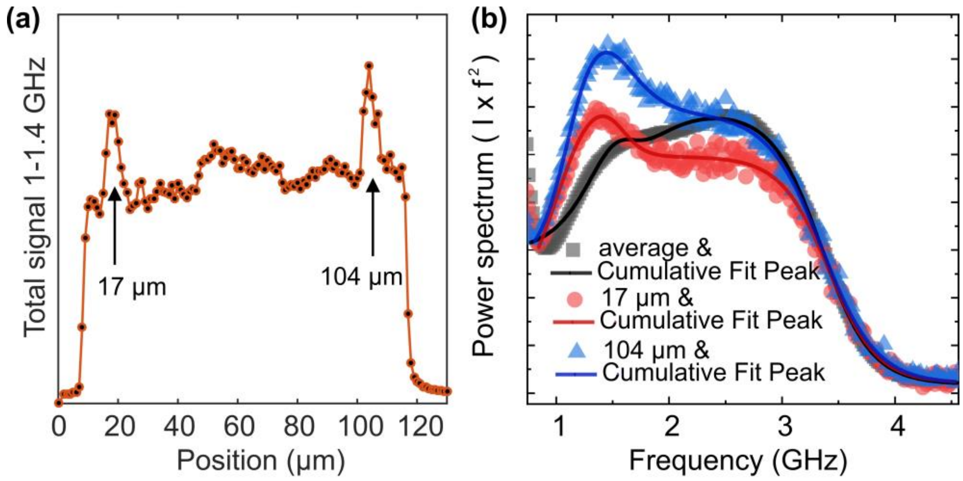

Figure S6: (a) The total intensity on the power spectrum of the HS (small particles, D=220 nm) integrated in the 1-1.4 GHz region. **(b)** The experimental power spectra (points) of HS in the 1-4 GHz range [the (1,1) mode] for the entire structure (grey), at 17 µm (red) and at 104 µm (blue) together with fittings of two asym2sig functions (same colour solid lines).

In **Figure S7**, the red curve shows the vibrational density of states for the FCC lattice, as calculated by standard numerical methods, assuming only nearest neighbor spring forces between nanoparticles. The spring force is $K_{eff} = \pi^2 M f_{11}^2$, where the mass $M = p\frac{4}{3}\pi\left(\frac{D}{2}\right)^3$, the

$D = 220$ nm the known particle diameter, and f_{11} the measured frequency of the (1,1) rattling mode. The phonon frequencies as a function of momentum are calculated on a uniform grid of 3·10[6] reciprocal space points. From the calculated band structure we then derive the vibrational density of states.

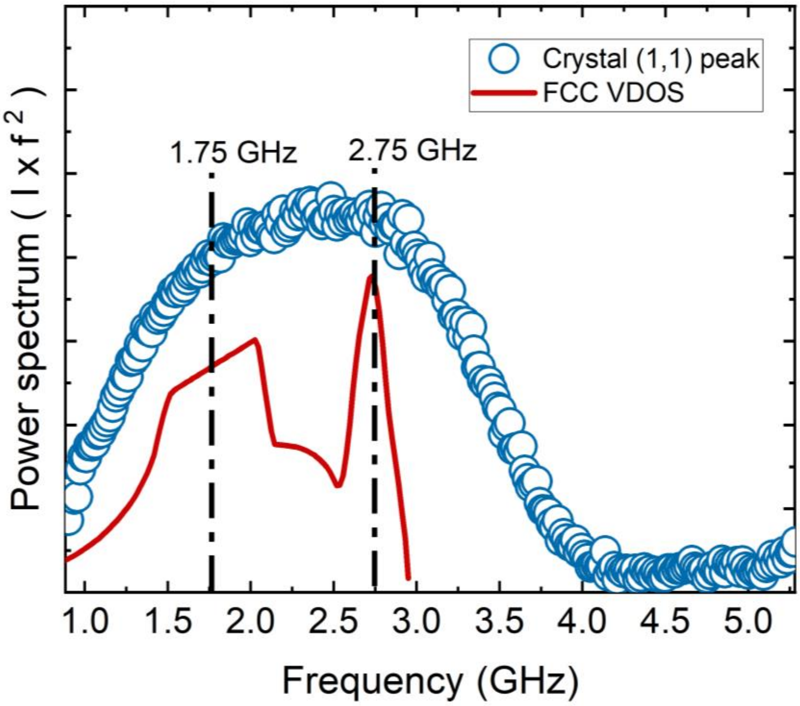

Figure S7: Comparison of the experimental power spectrum (blue points) of the crystalline edge of HS (main article, Figure 2e) with the calculated vibrational density of states of an FCC lattice with D=220 nm, lattice constant α=D, and spring constant, $K_{eff} = \pi^2 M f_{11}^2$.

S4: SEM imaging of the bilayer interface.

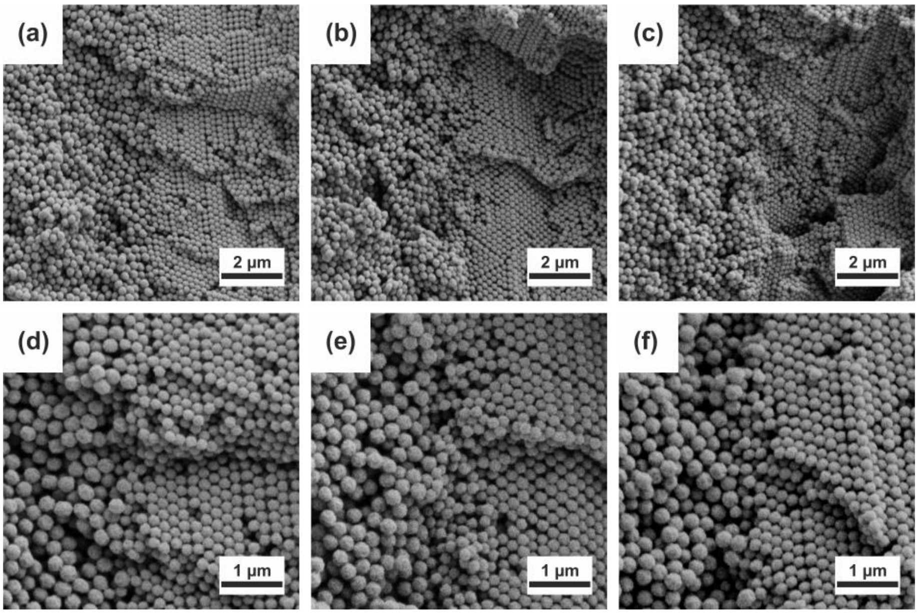

Figure S8 Characterization of the transition from large to small particles via scanning electron microscopy. a) – c) Overview images highlight transition region at various locations long the

colloidal sample. d) – f) Close-up characterization indicates a sharp transition from the large to the small particle side, which transitions within a few particle layers.

S5: Discussion on measurement and fitting uncertainties.

We calculate the error from the standard deviation of multiple measurements when available. For fitted quantities we provide the fitting uncertainty. For the calculated quantities we follow the standard procedure for error propagation. The error of the diameter D due to the measurement and fitting of the f_{12}' and f_{12}'' is $\delta D \approx 0.2\text{-}0.3$ nm. Averaged over many measurements the error is in the order of ~1 nm. There is however also error from the v_T, while the constant 0.84 is justified for spherical shapes. Regarding the v_T we have few measurements for single-sized samples and the standard deviation is ~20 m/s. This uncertainty increases the measurement errors for the particle diameters in the order of ± 5-7 nm or ~1.5-3%.

S6. Additional information on the gradient sample GOWR (220-450 nm).

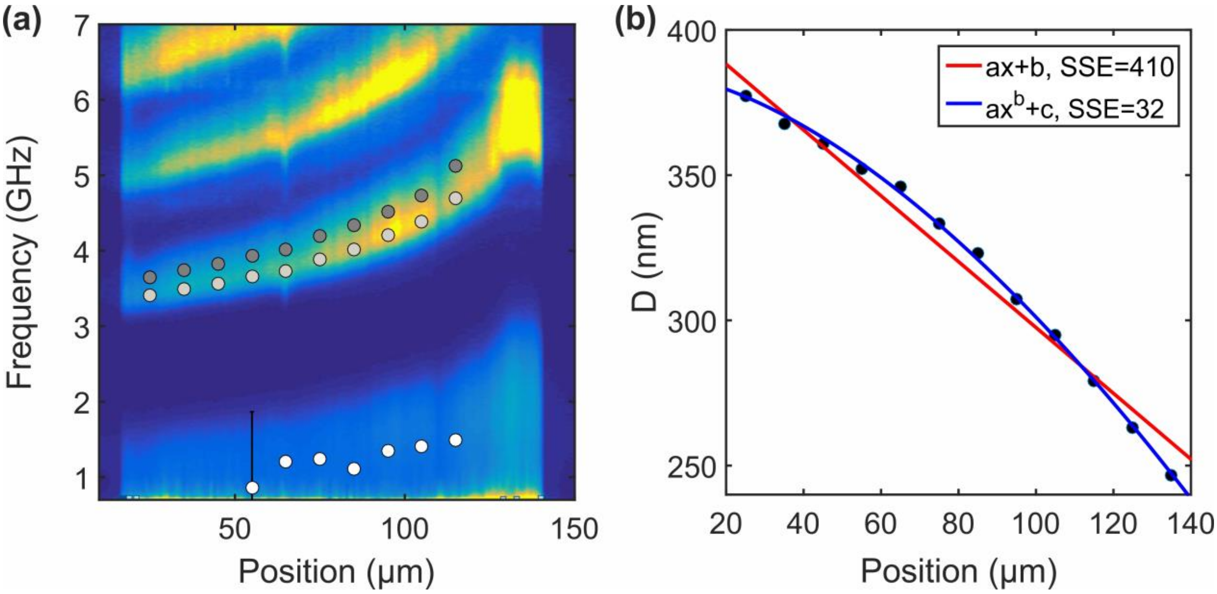

Figure S9: Elastic mapping of GOWR sample. (a) Spatial distribution of the power spectrum for the GOWR gradient structure, together with the fitting results for the frequencies of the (1,1) (white circles) and (1,2) f12' (grey) and f12'' (dark greay) mode (red) represented by an asym2sig spectral shape and a double Gaussian peak profile, respectively. **(b)** The particle diameter as a function of the position for the gradient structure. The measured D (points) is represented by either a linear function (red solid line) or a nonlinear function (blue solid line), with the latter giving significantly smaller sum of square errors (SSE). Thus, the GOWR has a spatially nonlinear distribution of sizes, in contrast to the mostly linear sample GO.

S7. Size-dependent effective elastic constant for all samples.

Figure S10 compares the E_{eff} between various samples at regions having locally the same particle size. The error bars represent the range of values of the filling factor. **Figure S11a** shows the experimental f_{11} frequencies of the gradient ordered samples, GO and GOWR, as a function of the local diameter D of the particles (points, red for GO and dark red for GOWR). The experimental data are represented with a power function of the form: aD^{-n}. The fitted exponent value, which is also noted in the diagram, is $-n = -(2.0 \pm 0.1)$. In **Figure S11b** we plot the E_{eff} as a function of D for all samples. For the sample corresponding to each experimental data set, see the same-color notes in the diagram. The error bars represent the uncertainty introduced by the filling factor $a \in [0.6, 0.74]$, which enters the formula for E_{eff}. We see that despite the uncertainty regarding the filling factor, the samples follow a clear trend: (i) spatially homogeneous single-sized samples have higher E_{eff} than the other samples at approximately the same D, (ii) spatially homogeneous bidisperse and polydisperse samples have lower E_{eff} than other samples at approximately the same D, and (iii) the gradient ordered samples lie in the middle of the other two regions. **Figure S11c** shows the same analysis for the effective spring constant K_{eff}, which is given by a relationship that does not depend explicitly on the filling factor. This time the error bars represent mainly the uncertainty introduced by calculating the mass as a function of the diameter, with $M \propto D^3$.

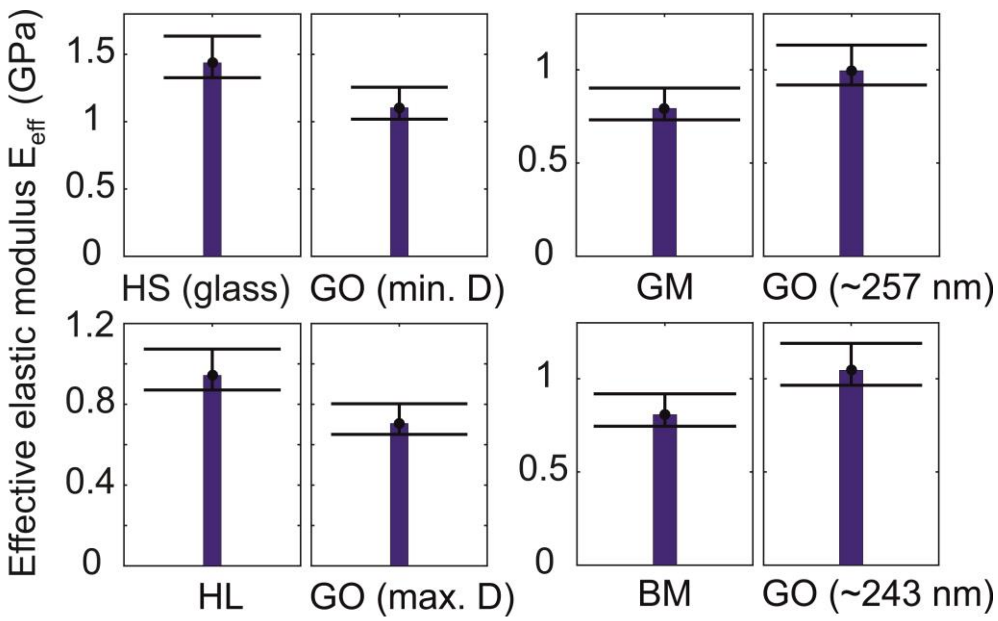

Figure S10: Comparison of the effective elastic moduli for homogeneous (HS), gradient ordered (GO), gradient mixed (GM), and binary mixed (BM) samples at regions where the local diameter is the same.

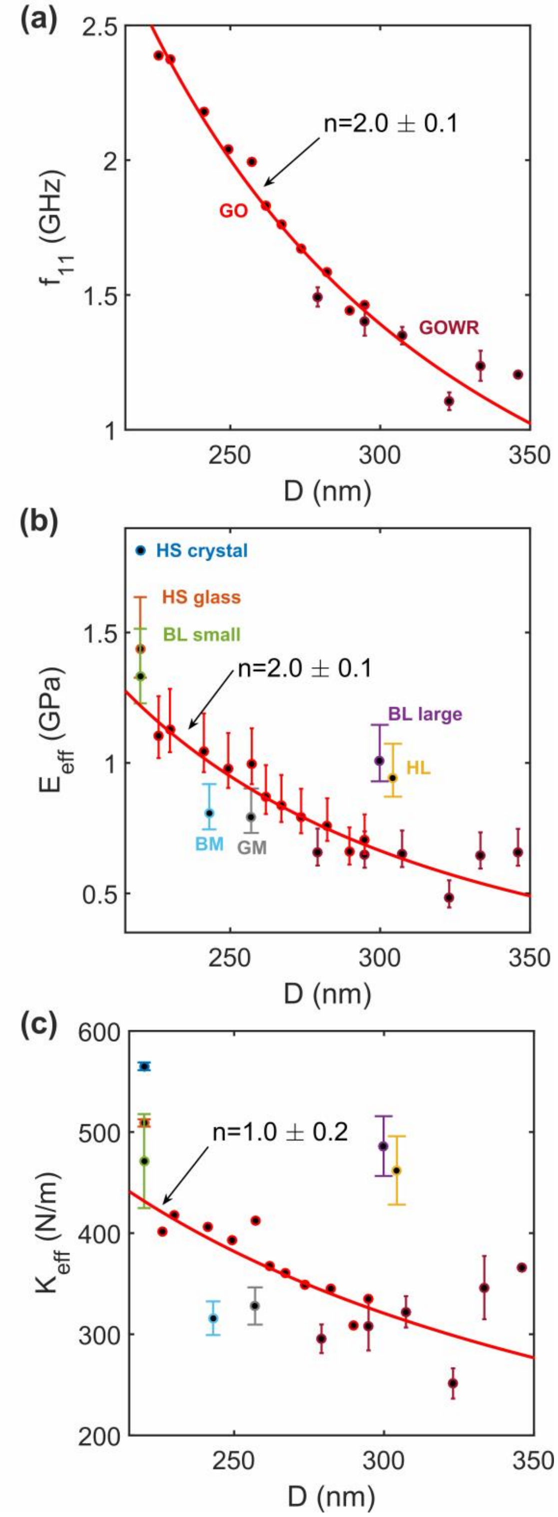

Figure S11: The f_{11} frequency **(a)**, the effective elastic constant E_{eff} **(b)**, and the effective spring constant K_{eff} **(c)**, as a function of the particle diameter D for all samples. When the (1,1) mode has a double peak (pure 220 nm and small particles' side of bilayer samples) the calculation of E_{eff} is performed with the highest frequency. The red solid line is a representation of the GO and GOWR data with a function of the form aD^{-n} with the fitted value of n given in each graph. In **(a)** the error bards represent the fitting uncertainty of f_{11}. In **(b)** the error bars give the interval of E_{eff} when the volume fraction α varies in the interval 0.6 to 0.74 with the central point being $a = 0.65$. In **(c)** the error bars show the uncertainty of K_{eff} due to both the f_{11} and the particle mass $M \propto D^3$.

S8: Frequency of (1,1) mode in binary mixed (BM) samples.

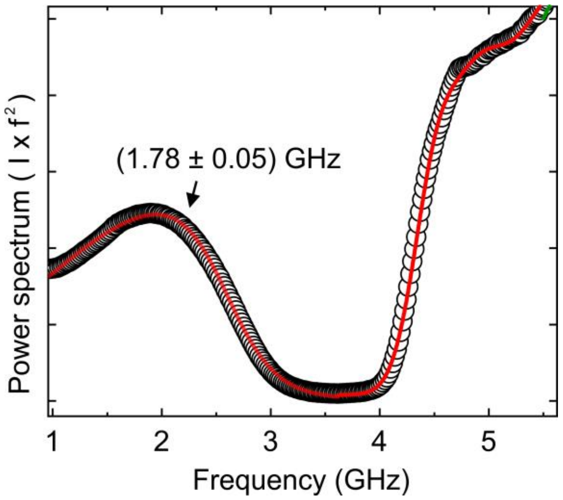

Figure S12: The experimental power spectrum (circles) of the mixed gradient sample GM with the (1,1) mode represented with an asym2sig function with peaked at 1.78 GHz plus a representation of the quadrupolar modes (red solid line) with Gaussian peaks. The signal above 4 GHz is due to a continuum of nanoparticle sizes starting from D=310 nm (f_{red}=3.93 GHz) that is represented by three Gaussian peaks.
